# Summary of best evidence for nutritional management in adult patients undergoing continuous renal replacement therapy

**DOI:** 10.3389/fmed.2026.1749845

**Published:** 2026-02-20

**Authors:** Liwei Jia, Jiangnan Wei, Kang Zhu, Xue Wang, Yuejuan Sun

**Affiliations:** Emergency Department (Xiangjiang Campus), Hebei Medical University Third Hospital, Shijiazhuang, Hebei, China

**Keywords:** blood purification, continuous renal replacement therapy, evidence summary, evidence-based nursing, nutrition

## Abstract

**Objective:**

To retrieve, screen, appraise, and synthesize the best evidence on nutritional management for adult patients undergoing continuous renal replacement therapy (CRRT) to inform the development of standardized clinical nutrition management protocols for CRRT.

**Methods:**

Using the 5S evidence pyramid model, we conducted a top-down search of domestic and international sources, including databases of systematic reviews, guideline portals, professional association websites, and comprehensive databases, to identify clinical decision aids, evidence summaries, guidelines, standards, protocols, systematic reviews, and expert consensus statements. The search period was from January 1, 2006, to December 31, 2025. Two evidence-based nursing experts independently performed quality appraisal, evidence extraction, and grading for the included literature.

**Results:**

A total of 12 documents were included: one clinical decision aid, one evidence summary, eight guidelines, and two expert consensus statements. After systematic extraction and integration of the relevant evidence, 16 evidence points were summarized across three domains: nutritional assessment and selection of nutritional pathways, energy and protein provision, and monitoring and supplementation of electrolytes and micronutrients.

**Conclusion:**

The best available evidence on nutritional management for adults undergoing CRRT synthesized in this study is comprehensive and scientifically sound. It provides a reference for clinical practice. However, personalized nutritional plans should be developed based on patients' clinical conditions to improve outcomes.

## 1 Introduction

The incidence of acute kidney injury (AKI) in intensive care units (ICUs) reaches 30%−60% (1–4). Continuous renal replacement therapy (CRRT) is a modality that continuously and slowly removes excess fluid and solutes and has been widely used in ICUs (5–7). Patients undergoing CRRT often have concomitant multi-organ injury and frequently exhibit hypercatabolism (2, 8). Furthermore, while CRRT removes excess fluid and solutes, it also eliminates small-molecule nutrients such as amino acids, short peptides, glucose, and vitamins (9–12). Current research (12–14) suggests that nutritional loss during CRRT plays a significant role in the progression of malnutrition in AKI (15, 16). Existing evidence syntheses on CRRT predominantly focus on anticoagulation strategies and operational management (17, 18), and optimal evidence for nutritional management during CRRT remains limited. Therefore, this study synthesizes the best available evidence on nutritional management for CRRT patients from domestic and international sources to inform nutritional care for this population. This study was registered with the Evidence-Based Nursing Center at Fudan University (ES20246481).

## 2 Data and methods

### 2.1 Evidence-based question formulation

We used the PIPOST framework (19) to define the evidence question: (1) population (P): adults (≥18 years) undergoing CRRT; (2) intervention (I): nutritional assessment, enteral and parenteral nutrition, and nutritional monitoring during CRRT; (3) professional (P): healthcare professionals; (4) outcomes (O): nutritional status, serum albumin; (5) setting (S): intensive care unit (ICU); and (6) type of evidence (T): guidelines, evidence summaries, systematic reviews, expert consensus statements, clinical decision aids, or recommended practices.

### 2.2 Search strategy

Two researchers trained in evidence-based methodology developed search terms and strategies, which were reviewed by an evidence-based nursing expert. Following the “5S” classification model of evidence-based search resources (20), we searched using a combination of subject headings and free-text terms. Databases searched included Embase, Web of Science, PubMed, the Cumulative Index to Nursing and Allied Health Literature (CINAHL), the Cochrane Library, China Biology Medicine (CBM), China National Knowledge Infrastructure (CNKI), Wanfang Data, VIP, the Joanna Briggs Institute (JBI) Evidence-Based Healthcare Library, UpToDate, and BMJ resources. Guideline websites included the World Health Organization (WHO), the National Guideline Clearinghouse (NGC), the National Institute for Health and Care Excellence (NICE), Kidney Disease: Improving Global Outcomes (KDIGO), the Scottish Intercollegiate Guidelines Network (SIGN), the Guidelines International Network (GIN), the New Zealand Guidelines Group (NZGG), and the Registered Nurses' Association of Ontario (RNAO). Professional association websites included the Chinese Society for Parenteral and Enteral Nutrition, the National Kidney Foundation, the International Society of Nephrology, the Chinese Society of Nephrology, the American Society for Parenteral and Enteral Nutrition, the European Society for Clinical Nutrition and Metabolism, the Society of Critical Care Medicine, and the European Society of Intensive Care Medicine. The search period was from January 1, 2006, to December 31, 2025. Relevant search terms are provided in Supplementary material S1.

### 2.3 Inclusion and exclusion criteria for literature

Inclusion criteria: (1) adults (≥18 years) undergoing CRRT; (2) studies involving nutritional assessment, intervention, or monitoring during CRRT; (3) language: Chinese or English; and (4) evidence type: clinical decision aids, guidelines, evidence summaries, systematic reviews, expert consensus statements, best practices, recommended practices, or standards.

Exclusion criteria: (1) duplicate publications; (2) full text unavailable; (3) translated versions; and (4) low methodological quality.

### 2.4 Quality appraisal

Clinical decision aids were considered high-level evidence and directly included. Evidence summaries were appraised using the Critical Appraisal for Summaries of Evidence (CASE) tool (21), which comprises 10 items rated as Yes, Partially, or No. Guidelines were appraised with the Appraisal of Guidelines for Research and Evaluation II (AGREE II) instrument (22). The JBI critical appraisal tools (23) and an expert consensus appraisal tool (24) were used to assess the methodological quality of the included systematic reviews and expert consensus statements, respectively. Two researchers independently conducted all appraisals in duplicate; disagreements were resolved by the corresponding author.

### 2.5 Evidence extraction, synthesis, and grading

Two researchers (Liwei Jia, Kang Zhu) independently screened records and extracted data using a customized form capturing inclusion criteria, publication date, source, type, and topic. A third evidence-based nursing expert (Xue Wang) verified semantics and content. When translation-related or cultural adaptation discrepancies arose, team members with professional English backgrounds (Jiangnan Wei, Xue Wang, Yuejuan Sun) discussed and reached consensus. During synthesis, concordant evidence was summarized; complementary evidence was logically integrated; in cases of conflict, higher-level, higher-quality, and more recent sources were prioritized. The synthesized evidence was graded using the JBI grades of evidence and levels of recommendation (2014) (25), with evidence levels from 1 to 5.

## 3 Results

### 3.1 Characteristics of included literature

We initially identified 2,097 records. After removing 952 duplicates, we screened titles, abstracts, and full texts. Following the exclusion of 1,133 records that did not meet the inclusion criteria, 12 studies were included. Their general characteristics are summarized in Table 1.

**Table 1:** Basic characteristics of included literature (*n* = 12).

**Included studies**	**Publication year (year)**	**Document type**	**Source**	**Document topic**
Connor (26)	2024	Clinical decision making	Uptodate	Prescription for continuous renal replacement therapy in adult acute kidney
Liu Jialong (18)	2024	Evidence summary	CNKI	Summary of best evidence for the management of continuous renal replacement therapy in adults with acute kidney injury
Sabatino (27)	2024	Guideline	ESPEN	ESPEN practical guideline on clinical nutrition in hospitalized patients with acute or chronic kidney disease
Chinese society of critical care medicine (28)	2023	Guideline	Chinese Medical Journal Full-text Database	Clinical practice guidelines for nutritional assessment and monitoring of adult ICU patients in China
National clinical research center for kidney disease (29)	2023	Guideline	China Medical Journal Full-text Database	Chinese clinical practice guideline for acute kidney injury
NICE (30)	2018	Guideline	NICE	Renal replacement therapy and conservative management
Doi (31)	2016	GUIDELINE	Pubmed	The japanese clinical practice guideline for acute kidney injury 2016
Kanagasundaram (32)	2019	Guideline	Yimaitong	Clinical practice guideline acute kidney injury (AKI)
Zhejiang medical association critical care medicine branch (33)	2024	Expert consensus	Chinese Medical Journal Full-text Database	Expert consensus on clinical practice of parenteral nutrition therapy for critically ill patients in China
Hung (34)	2022	Expert consensus	Web Of Science	Nutrition support for acute kidney injury 2020-consensus of the taiwan AKI task force
Singer (35)	2023	Guideline	ESPEN	ESPEN practical and partially revised guideline: clinical nutrition in the intensive care unit
Chinese society of nephrology (36)	2022	Guideline	Chinese Medical Journal Full-text Database	Guidelines for the anticoagulant management of continuous renal replacement therapy

### 3.2 Quality appraisal results

#### 3.2.1 Guidelines

Eight guidelines were included. Results are shown in Table 2. The Appraisal of Guidelines for Research and Evaluation II (AGREE II) instrument was used, which comprises six domains with 23 items. Each item is scored from 1 to 7, where 1 indicates “strongly disagree” (completely noncompliant) and 7 indicates “strongly agree” (completely compliant). Domain scores were standardized using the formula: (actual score – minimum possible score)/(maximum possible score – minimum possible score) × 100%. Recommendation levels were defined as follows: if all six domains score ≥60%, the recommendation is grade A; if more than three domains score ≥30% and at least one domain scores < 60%, the recommendation is grade B; if more than three domains score < 30%, the recommendation is grade C.

**Table 2:** Quality assessment results of included guidelines (*n* = 8).

**Guideline**	**Percentage of standardization across various fields**						**≥60% number of fields (number)**	**≥30% number of fields (number)**	**Overall quality**
	**Scope and purpose**	**Stakeholder involvement**	**Rigor of development**	**Clarity of presentation**	**Applicability**	**Editorial independence**			
Sabatino (27)	97.2	68.0	84.4	90.3	48.9	79.2	5	6	B
Chinese society of critical care medicine (28)	84.7	72.2	78.6	80.6	72.9	85.4	6	6	A
National clinical research center for kidney diseases chinese nephrologist association (29)	88.9	59.5	64.8	83.3	46.9	92.9	4	6	B
Nice (30)	93.1	70.8	72.4	95.8	40.6	10.4	4	5	B
Doi (31)	72.2	55.6	38.5	88.9	73.8	16.7	3	5	B
Kanagasundaram (32)	100	88.89	78.30	93.03	68	93.18	6	6	A
Singer (35)	90.1	58.3	65.1	88.9	18.8	41.7	3	5	B
Chinese society of nephrology (36)	100	54.2	83.3	95.8	79.2	100	5	6	B

#### 3.2.2 Expert consensus statements

Two expert consensus documents met the quality standards and were included. The results are presented in Table 3.

**Table 3:** Quality assessment results of included expert consensus statements (*n* = 2).

**Included literature**	**Is the source of the viewpoint clearly indicated?**	**Does the viewpoint originate from influential experts in the field?**	**Is the proposed viewpoint centered on the interests of the relevant population?**	**Are the stated conclusions based on analytical results? Is the expression of the viewpoint logical?**	**Has existing literature been referenced?**	**Are there inconsistencies between the proposed viewpoint and previous literature?**
Zhejiang medical association critical care medicine branch (33)	Yes	Yes	Yes	Yes	Yes	Not sure
Hung (34)	Yes	Yes	Yes	Yes	Yes	Yes

#### 3.2.3 Clinical decision aid

One clinical decision aid was included (26), defaulting to high-level evidence.

#### 3.2.4 Quality assessment of evidence summaries

In addition, we included one evidence summary (18), the evaluation results were as follows: item 3, “Transparency of reviewers or editors,” and item 5, “Transparency and translatability of the evidence grading system,” were rated “No”; item 7, “Appropriate citation of recommendations,” was rated “Not entirely”; all other items were rated “Yes.” This summary was therefore included.

After systematically extracting and integrating the relevant evidence, 16 evidence points were summarized across three domains: nutritional assessment and selection of nutritional pathways, energy and protein provision, and monitoring and supplementation of electrolytes and micronutrients (see Table 4).

**Table 4:** Summary of best evidence for nutritional management in adults undergoing continuous renal replacement therapy.

**Topic**	**Evidence content**	**Evidence level**
Nutritional assessment and selection of nutritional pathways	1 Patients with acute kidney injury (AKI) who are candidates for renal replacement therapy should be referred to a dietitian for individualized assessment (30, 32).	1d
	2 It is recommended that enteral nutrition be prioritized as the primary method of nutritional support for patients with acute kidney injury undergoing continuous renal replacement therapy (29, 32).	1b
	3 In patients deemed to be at high risk for aspiration, postpyloric, mainly jejunal feeding can be performed (35).	2d
Energy and protein provision	4 The administration of calorie and protein as nutritional support for AKI treatment be tailored to the severity and the underlying disease (31).	2d
	5 Patients with AKI undergoing CRRT typically present with other severe comorbidities. Early enteral nutrition is recommended within 48 h. When enteral pathways cannot meet >60% of energy and protein requirements within 7–10 days, parenteral nutrition should be initiated. Full-energy nutrition is not recommended during the acute phase of illness. Post-acute phase (after 72 h), energy intake may be gradually increased to achieve feeding targets of 80%−100% (18, 34).	2c
	6 It is recommended that the total energy intake for CRRT patients with AKI be maintained at 20–30 kcal/kg/day. When feasible, indirect calorimetry may be employed during CRRT intervals to assess energy expenditure (27–29, 34).	2c
	7 For patients undergoing KRT, the total energy provision by additional calories given in the form of citrate, lactate, and glucose from dialysis/hemofiltration solutions should be included in the calculations to determine the total daily energy provision to avoid overfeeding (37).	2b
	8 For patients undergoing CRRT, protein intake should be 1.5–1.7 g/kg/day. If necessary, it may be increased to 2.5 g/kg/day (18, 31, 33, 34).	2c
	9 Protein intake should not be restricted in order to delay the initiation of CRRT (27).	2c
Monitoring and supplementation of electrolytes and micronutrients	10 Electrolyte abnormalities are common in critically ill 11 patients undergoing CRRT and should be closely monitored (28, 37).	1a
	12 Initially, monitor electrolytes and acid-base status every 6–12 h. If the patient remains stable within 24–48 h with minimal electrolyte changes, the frequency of electrolyte testing may be reduced to every 12–24 h (18, 26).	2d
	13 During local citrate anticoagulation, two calcium ion concentrations require monitoring: maintaining extracorporeal calcium ion levels between 0.25 and 0.40 mmol/L achieves optimal local anticoagulation; Intracorporeal calcium ion levels should be maintained within the normal physiological range of 1.1–1.3 mmol/L (18, 36).	2c
	14 Due to increased requirements during AKI and critical illness, as well as substantial fluid losses during CRRT, trace elements should be monitored and supplemented. Increased attention should be given to selenium, zinc, and copper (27).	1b
	15 Due to increased requirements during AKI and critical illness, as well as substantial fluid losses during CRRT, water-soluble vitamins should be monitored and supplemented. Particular attention should be paid to vitamin C, folic acid, and thiamine (27).	1b
	16 Dialysate containing potassium, phosphate, and magnesium should be used to prevent electrolyte disturbances during CRRT (27).	1c

## 4 Discussion

### 4.1 Calorie and protein loss and replacement

#### 4.1.1 Calorie expenditure and replacement

Since CRRT does not alter the energy requirements of patients with AKI, current guidelines recommend an energy intake of 20–30 kcal/kg/day across all stages of AKI (27–29, 38). Indirect calorimetry is considered the gold standard for measuring resting energy expenditure (REE) in critically ill patients (39) and may be used to assess actual energy consumption when conditions permit. Multiple studies (40, 41) indicate that non-nutritive calories (NNCs) from propofol, glucose, and citrate in the CRRT circuit can contribute meaningfully to total energy intake. However, a retrospective study of 33 patients undergoing CVVHD (42) found that, in non-hyperglycemic patients, the metabolic contributions of lactate, glucose, and citrate were negligible, whereas in hyperglycemic patients these substrates were associated with substantial caloric loss (up to approximately 600 kcal/day). This finding may relate to the CRRT modality and patients' baseline hyperglycemia, which can increase glucose losses during therapy. Patients receiving CRRT are typically critically ill with multi-organ injury, and their energy requirements are closely linked to the underlying disease. Therefore, in clinical practice, energy provision should be individualized based on disease status, exogenous caloric intake and losses, and the CRRT modality.

#### 4.1.2 Protein loss and replacement

Protein-energy wasting (PEW) is highly prevalent in patients with acute kidney injury (AKI) and is associated with prolonged hospitalization, increased complications, and higher mortality. Non-selective solute removal during CRRT may exacerbate PEW. During CRRT, various amino acids are removed (43); the degree of loss correlates with the patient's clinical condition and treatment modality, with CVVH demonstrating the greatest amino acid clearance (9, 10). Tatsumi's study (44) shows that in AKI patients without nutritional supplementation, blood amino acid concentrations remained stable during CVVH, yet substantial amounts of amino acids were detected in the effluent, potentially reflecting endogenous protein catabolism. Different studies (44, 45) estimate amino acid losses ranging from 5.7 to 13.4 g, which may relate to differences in membrane type, exogenous amino acid supplementation, and baseline serum amino acid concentrations. The ESPEN guidelines (27) recommend a protein intake of 1.5–1.7 g/kg/day for patients undergoing CRRT, whereas Hung et al. (34) recommend 1.5–2.5 g/kg/day. Research by van Ruijven et al. (46) found that early high-protein intake (≥1.2 g/kg/day) in patients receiving CRRT was significantly associated with lower hospital and ICU mortality. However, evidence linking higher protein intake to improved clinical outcomes remains limited.

### 4.2 Monitoring and supplementation of electrolytes and micronutrients

#### 4.2.1 Monitoring and replenishment of electrolytes

Hypomagnesemia is quite common in AKI patients undergoing CRRT, with its incidence varying depending on the CRRT modality, treatment dosage, anticoagulation strategy, and composition of the replacement fluid/dialysate (47–49). The specific mechanisms of magnesium loss remain incompletely understood, potentially involving direct loss through filters and chelation with citrate in the extracorporeal circulation (50). However, this mechanism has not been confirmed in subsequent studies. Current guidelines recommend supplementing magnesium in the dialysate, but the optimal magnesium concentration for different CRRT protocols has yet to be established. Hypophosphatemia occurs in 54%−85% of critically ill patients undergoing CRRT (11); this may result in ventilator failure, difficulty weaning off the ventilator, and arrhythmias (11, 51). The occurrence of hypophosphatemia in CRRT patients may be associated with nonspecific clearance during the CRRT process. The use of phosphate-containing CRRT solutions is a safe and effective core strategy for preventing CRRT-induced hypophosphatemia. Combined with daily serum phosphorus monitoring, individualized phosphorus supplementation (oral or intravenous), and avoidance of electrolyte disturbances, this approach can significantly improve patient outcomes (52).

Regional citrate anticoagulation has become the preferred anticoagulation method for CRRT treatment in patients without contraindications due to its low bleeding risk (36, 53, 54). Because it relies on citrate to chelate calcium ions in the blood to inhibit activation of the coagulation system, it may cause disturbances in the patient's acid-base balance and fluctuations in blood calcium levels during treatment. Multiple studies (55, 56) have confirmed that ionized calcium levels in CRRT patients are closely associated with prognosis, with hypocalcemia increasing the risk of adverse outcomes. Current guidelines recommend monitoring electrolytes and acid-base status every 6–12 h initially for CRRT patients receiving citrate anticoagulation. If the patient remains stable within 24–48 h, electrolyte testing frequency may be reduced to every 12–24 h. Maintaining extracorporeal calcium ion concentration at 0.25–0.40 mmol/L achieves effective local anticoagulation, while maintaining intravascular calcium ion concentration within the normal physiological range of 1.1–1.3 mmol/L (36).

#### 4.2.2 Micronutrient monitoring and supplementation

Micronutrients play a central role in numerous metabolic processes and cellular functions (38, 57). However, due to diffusion or adsorption, vitamins and trace elements may experience additional losses during CRRT treatment. The extent of these losses varies depending on the CRRT method, dosage, duration, and the specific type of micronutrient involved (38). Vitamin C, folic acid, selenium, copper, zinc, and carnitine can be detected in the effluent, with vitamin C and carnitine exhibiting the most significant losses (12, 58, 59). Liposoluble vitamins (A, D, E, K) and certain water-soluble vitamins (B1, B6, B12, potentially due to dilution, metabolic conversion, or adsorption) were not detected in the filtrate, yet their plasma concentrations showed a significant decrease, possibly related to adsorption by the membrane (9). The concentrations of trace elements such as selenium, zinc, and copper in the filtrate vary, potentially influenced by the patient's clinical condition, CRRT mode, and duration. A study involving 50 adult patients (60) demonstrated that vitamin B6, vitamin C, and folate levels significantly decreased 72 h after initiating CRRT, consistent with findings reported by Fah et al. (59). It is important to emphasize the monitoring and supplementation of micronutrients. Unfortunately, due to variations in nutritional delivery methods across studies, the relatively short duration of CRRT, and small sample sizes, it is not possible to provide dosage and regimen recommendations for nutritional supplementation in clinical practice (38, 61).

Summary: this study synthesizes the best available evidence on nutritional management during continuous renal replacement therapy (CRRT) in adults, providing an evidence-based foundation for clinical practice. Healthcare providers should develop personalized nutritional treatment plans tailored to each patient's disease status, CRRT modality, and treatment duration. As most included literature is in English, applying evidence should be contextualized to clinical settings. Future research should explore the effects of different CRRT modes, materials, and durations on caloric and nutrient requirements in AKI patients, thereby providing more reliable guidance for nutritional management in this population.
