# Supplementary material for: Summary of best evidence for nutritional management in adult patients undergoing continuous renal replacement therapy
Source: Front Med (Lausanne). 2026 Feb 20;13:1749845. doi: 10.3389/fmed.2026.1749845 (PMC12964707; doi:10.3389/fmed.2026.1749845)
Supplement: Supplementary file 1 [file Data_Sheet_1.docx]

**Table S1** Search strategy

1. **Pubmed**

| Search number | Query | Results |
| --- | --- | --- |
| #9 | Time Limit: 2006 – 2026 | 276 |
| #8 | #3 AND #7 | 408 |
| #7 | #4 OR #5 OR #6 | 341999 |
| #6 | ((Support, Nutritional[Title/Abstract]) OR (Artificial Feeding[Title/Abstract])) OR (Feeding, Artificial[Title/Abstract]) | 1376 |
| #5 | (((Therapy, Nutrition[Title/Abstract]) OR (Medical Nutrition Therapy[Title/Abstract])) OR (Nutrition Therapy, Medical[Title/Abstract])) OR (Therapy, Medical Nutrition[Title/Abstract]) | 1289 |
| #4 | (nutrition therapy) OR (nutritional support) | 341114 |
| #3 | #1 OR #2 | 8984 |
| #2 | ((((((((((((((((((((((((((((((((((((((((((((((((((CRRT Technique[Title/Abstract]) OR (Continuous Renal Replacement Procedure[Title/Abstract])) OR (Continuous RRT[Title/Abstract])) OR (Continuous RRTs[Title/Abstract])) OR (RRT, Continuous[Title/Abstract])) OR (Hemofiltration, Continuous Arteriovenous[Title/Abstract])) OR (Arteriovenous Hemofiltration, Continuous[Title/Abstract])) OR (Continuous Arteriovenous Hemofiltration[Title/Abstract])) OR (Continuous Arteriovenous Hemofiltrations[Title/Abstract])) OR (Continuous Arterio-Venous Ultrafiltration[Title/Abstract])) OR (Arterio-Venous Ultrafiltration, Continuous[Title/Abstract])) OR (Continuous Arterio Venous Ultrafiltration[Title/Abstract])) OR (Continuous Arterio-Venous Ultrafiltrations[Title/Abstract])) OR (Ultrafiltration, Continuous Arterio-Venous[Title/Abstract])) OR (CAVHD[Title/Abstract])) OR (Continuous Venovenous Hemodiafiltration[Title/Abstract])) OR (Continuous Venovenous Hemodiafiltrations[Title/Abstract])) OR (Hemodiafiltration, Continuous Venovenous[Title/Abstract])) OR (Venovenous Hemodiafiltration, Continuous[Title/Abstract])) OR (Continuous Veno-Venous Hemodiafiltration[Title/Abstract])) OR (Continuous Veno Venous Hemodiafiltration[Title/Abstract])) OR (Continuous Veno-Venous Hemodiafiltrations[Title/Abstract])) OR (Hemodiafiltration, Continuous Veno-Venous[Title/Abstract])) OR (Veno-Venous Hemodiafiltration, Continuous[Title/Abstract])) OR (CVVHDF[Title/Abstract])) OR (Continuous Venovenous Hemodialysis[Title/Abstract])) OR (Continuous Venovenous Hemodialyses[Title/Abstract])) OR (Hemodialysis, Continuous Venovenous[Title/Abstract])) OR (Venovenous Hemodialysis, Continuous[Title/Abstract])) OR (Continuous Veno-Venous Hemodialysis[Title/Abstract])) OR (Continuous Veno-Venous Hemodialyses[Title/Abstract])) OR (Continuous Veno Venous Hemodialysis[Title/Abstract])) OR (Hemodialysis, Continuous Veno-Venous[Title/Abstract])) OR (Veno-Venous Hemodialyses, Continuous[Title/Abstract])) OR (Veno-Venous Hemodialysis, Continuous[Title/Abstract])) OR (CVVHD[Title/Abstract])) OR (Continuous Venovenous Hemofiltration[Title/Abstract])) OR (Continuous Venovenous Hemofiltrations[Title/Abstract])) OR (Hemofiltration, Continuous Venovenous[Title/Abstract])) OR (Venovenous Hemofiltration, Continuous[Title/Abstract])) OR (CVVH Technique[Title/Abstract])) OR (CVVH Techniques[Title/Abstract])) OR (Continuous Veno-Venous Hemofiltration[Title/Abstract])) OR (Continuous Veno Venous Hemofiltration[Title/Abstract])) OR (Continuous Veno-Venous Hemofiltrations[Title/Abstract])) OR (Hemofiltration, Continuous Veno-Venous[Title/Abstract])) OR (Veno-Venous Hemofiltration, Continuous[Title/Abstract])) OR (Slow Continuous Ultrafiltration[Title/Abstract])) OR (Slow Continuous Ultrafiltrations[Title/Abstract])) OR (SCUF Technique[Title/Abstract])) OR (SCUF Techniques[Title/Abstract]) | 2838 |
| #1 | "continuous renal replacement therapy" | 7419 |

1. **web of science（core collection）**

| Search number | Query | Results |
| --- | --- | --- |
| #3 | #1 AND #2 | 46 |
| #2 | “nutrition therapy” OR “Therapy, Nutrition” OR “Medical Nutrition Therapy” OR “Nutrition Therapy, Medical” OR “Therapy, Medical Nutrition” OR “Nutritional Support” OR “Support, Nutritional” OR “Artificial Feeding” OR “Feeding, Artificial”(Time Limit: 2006-01-01 – 2026-01-01) | 12222 |
| #1 | “continuous renal replacement therapy” OR “CRRT Technique” OR “Continuous Renal Replacement Procedure” OR “Continuous RRT” OR “Continuous RRTs” OR “RRT, Continuous” OR “Hemofiltration, Continuous Arteriovenous” OR “Arteriovenous Hemofiltration, Continuous” OR “Continuous Arteriovenous Hemofiltration” OR “Continuous Arteriovenous Hemofiltrations” OR “Continuous Arterio-Venous Ultrafiltration” OR “Arterio-Venous Ultrafiltration, Continuous” OR “Continuous Arterio Venous Ultrafiltration” OR “Continuous Arterio-Venous Ultrafiltrations” OR “Ultrafiltration, Continuous Arterio-Venous” OR “CAVHD” OR “Continuous Venovenous Hemodiafiltration” OR “Continuous Venovenous Hemodiafiltrations” OR “Hemodiafiltration, Continuous Venovenous” OR “Venovenous Hemodiafiltration, Continuous” OR “Continuous Veno-Venous Hemodiafiltration” OR “Continuous Veno Venous Hemodiafiltration” OR “Continuous Veno-Venous Hemodiafiltrations” OR “Hemodiafiltration, Continuous Veno-Venous” OR “Veno-Venous Hemodiafiltration, Continuous” OR “CVVHDF” OR “Continuous Venovenous Hemodialysis” OR “Continuous Venovenous Hemodialyses” OR “Hemodialysis, Continuous Venovenous” OR “Venovenous Hemodialysis, Continuous” OR “Continuous Veno-Venous Hemodialysis” OR “Continuous Veno-Venous Hemodialyses” OR “Continuous Veno Venous Hemodialysis” OR “Hemodialysis, Continuous Veno-Venous” OR “Veno-Venous Hemodialyses, Continuous” OR “Veno-Venous Hemodialysis, Continuous” OR “CVVHD” OR “Continuous Venovenous Hemofiltration” OR “Continuous Venovenous Hemofiltrations” OR “Hemofiltration, Continuous Venovenous” OR “Venovenous Hemofiltration, Continuous” OR “CVVH Technique” OR “CVVH Techniques” OR “Continuous Veno-Venous Hemofiltration” OR “Continuous Veno Venous Hemofiltration” OR “Continuous Veno-Venous Hemofiltrations” OR “Hemofiltration, Continuous Veno-Venous” OR “Veno-Venous Hemofiltration, Continuous” OR “Slow Continuous Ultrafiltration” OR “Slow Continuous Ultrafiltrations” OR “SCUF Technique” OR “SCUF Techniques”（Time Limit: 2006-01-01 – 2026-01-01） | 6630 |

3.EMBASE

| Search number | Query | Results |
| --- | --- | --- |
| #8 | #7(Time Limit:2006-01-01 – 2026-01-01) | 510 |
| #7 | #6 AND #3 | 537 |
| #6 | #4 OR #5 | 518817 |
| #5 | 'diet intervention':ti,ab,kw OR 'diet treatment':ti,ab,kw OR 'dietary intervention':ti,ab,kw OR 'dietary therapy':ti,ab,kw OR 'dietary treatment':ti,ab,kw OR 'dietotherapy':ti,ab,kw OR 'nutrition therapy':ti,ab,kw OR 'nutritional therapy':ti,ab,kw OR 'diet therapy':ti,ab,kw | 41127 |
| #4 | 'diet therapy'/exp | 504559 |
| #3 | #1 OR #2 | 21969 |
| #2 | 'ckrt':ti,ab,kw OR 'continuous kidney replacement therapy':ti,ab,kw OR 'continuous krt':ti,ab,kw OR 'continuous rrt':ti,ab,kw OR 'crrt':ti,ab,kw OR 'continuous renal replacement therapy':ti,ab,kw | 11107 |
| #1 | 'continuous renal replacement therapy'/exp | 19398 |

1. COCHRANE

| Search number | Query | Results |
| --- | --- | --- |
| #19 | #13 AND #18 | 14 |
| #18 | #14 OR #15 OR #16 OR #17 | 14727 |
| #17 | (Artificial Feeding):ti,ab,kw AND (Feeding, Artificial):ti,ab,kw AND (Support, Nutritional):ti,ab,kw | 127 |
| #16 | (Medical Nutrition Therapy):ti,ab,kw OR (Nutrition Therapy, Medical):ti,ab,kw | 1816 |
| #15 | MeSH descriptor: [Nutrition Therapy] explode all trees | 13180 |
| #14 | MeSH descriptor: [Nutritional Support] explode all trees | 4478 |
| #13 | #1 OR #2 OR #3 OR #4 OR #5 OR #6 OR #7 OR #8 OR #9 OR #10 OR #11 OR #12 | 1252 |
| #12 | (Ultrafiltration, Continuous Arterio-Venous):ti,ab,kw AND (Continuous Arteriovenous Hemofiltration):ti,ab,kw AND (Continuous Arterio-Venous Ultrafiltrations;):ti,ab,kw AND (CAVHD):ti,ab,kw AND (Continuous Arterio Venous Ultrafiltration):ti,ab,kw | 0 |
| #11 | (Hemofiltration, Continuous Arteriovenous):ti,ab,kw AND (Arterio-Venous Ultrafiltration, Continuous):ti,ab,kw AND (Continuous Arterio-Venous Ultrafiltration):ti,ab,kw AND (Arteriovenous Hemofiltration, Continuous):ti,ab,kw AND (Continuous Arteriovenous Hemofiltrations):ti,ab,kw | 0 |
| #10 | (Continuous RRT):ti,ab,kw AND (Continuous Renal Replacement Procedure):ti,ab,kw AND (CRRT Technique):ti,ab,kw AND (Continuous RRTs):ti,ab,kw AND (RRT, Continuous):ti,ab,kw | 0 |
| #9 | (Continuous Veno-Venous Hemodialyses):ti,ab,kw AND (Veno-Venous Hemodialyses, Continuous):ti,ab,kw AND (Hemodialysis, Continuous Venovenous):ti,ab,kw AND (Continuous Veno Venous Hemodialysis):ti,ab,kw AND (CVVHD):ti,ab,kw | 0 |
| #8 | (Continuous Veno-Venous Hemodialysis):ti,ab,kw AND (Hemodialysis, Continuous Veno-Venous):ti,ab,kw AND (Continuous Venovenous Hemodialysis):ti,ab,kw AND (Venovenous Hemodialysis, Continuous):ti,ab,kw AND (Veno-Venous Hemodialysis, Continuous):ti,ab,kw | 58 |
| #7 | (Hemodiafiltration, Continuous Veno-Venous):ti,ab,kw AND (Continuous Veno Venous Hemodiafiltration):ti,ab,kw AND (Hemodiafiltration, Continuous Venovenous):ti,ab,kw AND (Venovenous Hemodiafiltration, Continuous; Continuous Venovenous Hemodiafiltration):ti,ab,kw AND (Continuous Venovenous Hemodialyses):ti,ab,kw | 0 |
| #6 | (Continuous Venovenous Hemodiafiltrations):ti,ab,kw AND (CVVHDF):ti,ab,kw AND (Continuous Veno-Venous Hemodiafiltration):ti,ab,kw AND (Veno-Venous Hemodiafiltration, Continuous):ti,ab,kw AND (Continuous Veno-Venous Hemodiafiltrations):ti,ab,kw | 0 |
| #5 | (Continuous Venovenous Hemofiltration):ti,ab,kw AND (Hemofiltration, Continuous Venovenous):ti,ab,kw AND (CVVH Techniques):ti,ab,kw AND (Hemofiltration, Continuous Veno-Venous):ti,ab,kw AND (Continuous Venovenous Hemofiltrations):ti,ab,kw | 0 |
| #4 | (Venovenous Hemofiltration, Continuous):ti,ab,kw AND (Continuous Veno-Venous Hemofiltration):ti,ab,kw AND (Continuous Veno-Venous Hemofiltrations):ti,ab,kw AND (Continuous Veno Venous Hemofiltration):ti,ab,kw AND (CVVH Technique):ti,ab,kw | 0 |
| #3 | (Slow Continuous Ultrafiltration):ti,ab,kw OR (Slow Continuous Ultrafiltrations):ti,ab,kw OR (SCUF Technique):ti,ab,kw OR (SCUF Techniques):ti,ab,kw OR (Veno-Venous Hemofiltration, Continuous):ti,ab,kw | 159 |
| #2 | (Continuous Renal Replacement Therapy):ti,ab,kw | 1140 |
| #1 | MeSH descriptor: [Continuous Renal Replacement Therapy] explode all trees | 109 |

1. CINAHL

| Search number | Query | Results |
| --- | --- | --- |
| #3 | #1 AND #2 | 186 |
| #2 | SU (MH "Diet Therapy" OR MH "Nutrition" OR MH "Nutritive Value" OR MH "Nutritional Physiology" OR MH "Nutrition Disorders" OR MH "Nutritional Requirements" OR MH "Parenteral Nutrition" OR MH "Enteral Nutrition" OR MH "Prenatal Nutritional Physiology" OR MH "Parenteral Nutrition Solutions" OR MH "Nutritional Support Team" OR MH "Swallowing Therapy")（时间限制：2006-01-01---2026-01-01） | 115124 |
| #1 | [SU (MH "Continuous Renal Replacement Therapy" OR MH "Renal Replacement Therapy" OR MH "Renal Diet" OR MH "Continuous Venovenous Hemodialysis" OR MH "Continuous Venovenous Hemofiltration" OR MH "Continuous Venovenous Hemodiafiltration" OR MH "Slow Continuous Ultrafiltration")](https://research.ebsco.com/search/results?db=ccm,cul&expanders=concept&limiters=None&searchMode=boolean&sort=relevance&sqId=sq:58f0a5ee-6de2-412d-9c6f-f0b664d71506&userDirectAction=true)（Time Limit: 2006-01-01 – 2026-01-01） | 7530 |

1. Chinese Medical Journal Full-Text Database

| Search number | Query | Results |
| --- | --- | --- |
| #1 | SU = （Continuous renal replacement therapy OR Continuous veno-venous hemofiltration OR Continuous veno-venous hemodialysis OR Continuous veno-venous hemodiafiltration) AND SU = (Nutrition OR Nutritional intervention OR Nutritional therapy OR Enteral nutrition OR Parenteral nutrition OR Diet) (Time Limit: 2006–2026） | 445 |

1. CNKI

| Search number | Query | Results |
| --- | --- | --- |
| #1 | (SU = Continuous renal replacement therapy + Continuous veno-venous hemofiltration + Continuous veno-venous hemodialysis + Continuous veno-venous hemodiafiltration) AND (SU = Nutrition + Nutritional intervention + Nutritional therapy + Enteral nutrition + Parenteral nutrition + Diet) Exact match,（Time Limit: 2006-01-01---2026-01-01） | 44 |

1. China Biomedical Literature Database

| Search number | Query | Results |
| --- | --- | --- |
| #8 | #7（Time Limit: 2006-01-01---2026-01-01） | 384 |
| #7 | ((“Artificial Feeding”[Common Field: Smart] OR ‘Nutrition’[Common Field: Smart] OR “Nutritional Intervention”[Common Field: Smart] OR “Enteral Nutrition”[Common Field: Smart] OR “Parenteral Nutrition” [Common field: Smart] OR “diet”[Common field: Smart] OR “nutritional therapy”[Common field: Smart]) OR ((“nutritional support”[Unweighted: Expand]))) AND ((“continuous veno-venous hemodialysis”[Common field: Smart] OR “continuous veno-venous hemodiafiltration”[Common field: Smart] OR “slow continuous ultrafiltration” [Common Field: Smart] OR “Continuous Venous Hemofiltration”[Common Field: Smart]) OR (“Continuous Renal Replacement Therapy”[Unweighted: Expanded])) | 563 |
| #6 | (“Artificial Feeding”[Common Field: Smart] OR ‘Nutrition’[Common Field: Smart] OR “Nutritional Intervention” [Common field: Smart] OR “Enteral nutrition”[Common field: Smart] OR “Parenteral nutrition”[Common field: Smart] OR ‘Diet’[Common field: Smart] OR “Nutritional therapy”[Common field: Smart]) OR ((“Nutritional support”[Unweighted: Expanded])) | 896324 |
| #5 | “Artificial feeding”[Common field: Smart] OR “Nutrition”[Common field: Smart] OR “Nutritional intervention”[Common field: Smart] OR “Enteral nutrition”[Common field: Smart] OR “Parenteral nutrition”[Common field: Smart] OR ‘Diet’[Common field: Smart] OR “Nutritional therapy”[Common field: Smart] | 393646 |
| #4 | (“Nutritional Support”[unweighted:expanded]) | 39760 |
| #3 | #1 OR #2 | 11594 |
| #2 | (“Continuous Venous Hemodialysis”[Common Field: Smart] OR “Continuous Venous Hemodiafiltration”[Common Field: Smart] OR “Slow Continuous Ultrafiltration”[Common Field: Smart] OR “Continuous Venous Hemofiltration”[Common Field: Smart]) OR (“Continuous Renal Replacement Therapy”[Unweighted: Expanded]) | 9017 |
| #1 | Continuous renal replacement therapy | 5470 |

1. Wanfang Medical Network

| Search number | Query | Results |
| --- | --- | --- |
| #1 | (Subject=(Continuous renal replacement therapy OR Continuous veno-venous hemofiltration OR Continuous veno-venous hemodialysis OR Continuous veno-venous hemodiafiltration)) AND Subject=(Nutrition OR Nutritional intervention OR Nutritional therapy OR Enteral nutrition OR Parenteral nutrition OR Diet) (Time 2006--2025) | 347 |

1. VIP Database

| Search number | Query | Results |
| --- | --- | --- |
| #3 | #1 AND #2 | 154 |
| #2 | Title or Keywords=Nutrition OR Nutritional Intervention OR Nutritional Therapy OR Enteral Nutrition OR Parenteral Nutrition OR Diet (Synonym Expansion) (Year=2006-2025) | 343395 |
| #1 | Title or Keywords=Continuous renal replacement therapy OR Continuous veno-venous hemofiltration OR Continuous veno-venous hemodialysis OR Continuous veno-venous hemodiafiltration (synonym expansion) (Year=2006-2025) | 12600 |
