# Supplementary figures and images for: Summary of best evidence for nutritional management in adult patients undergoing continuous renal replacement therapy
Source: Front Med (Lausanne). 2026 Feb 20;13:1749845. doi: 10.3389/fmed.2026.1749845 (PMC12964707; doi:10.3389/fmed.2026.1749845)

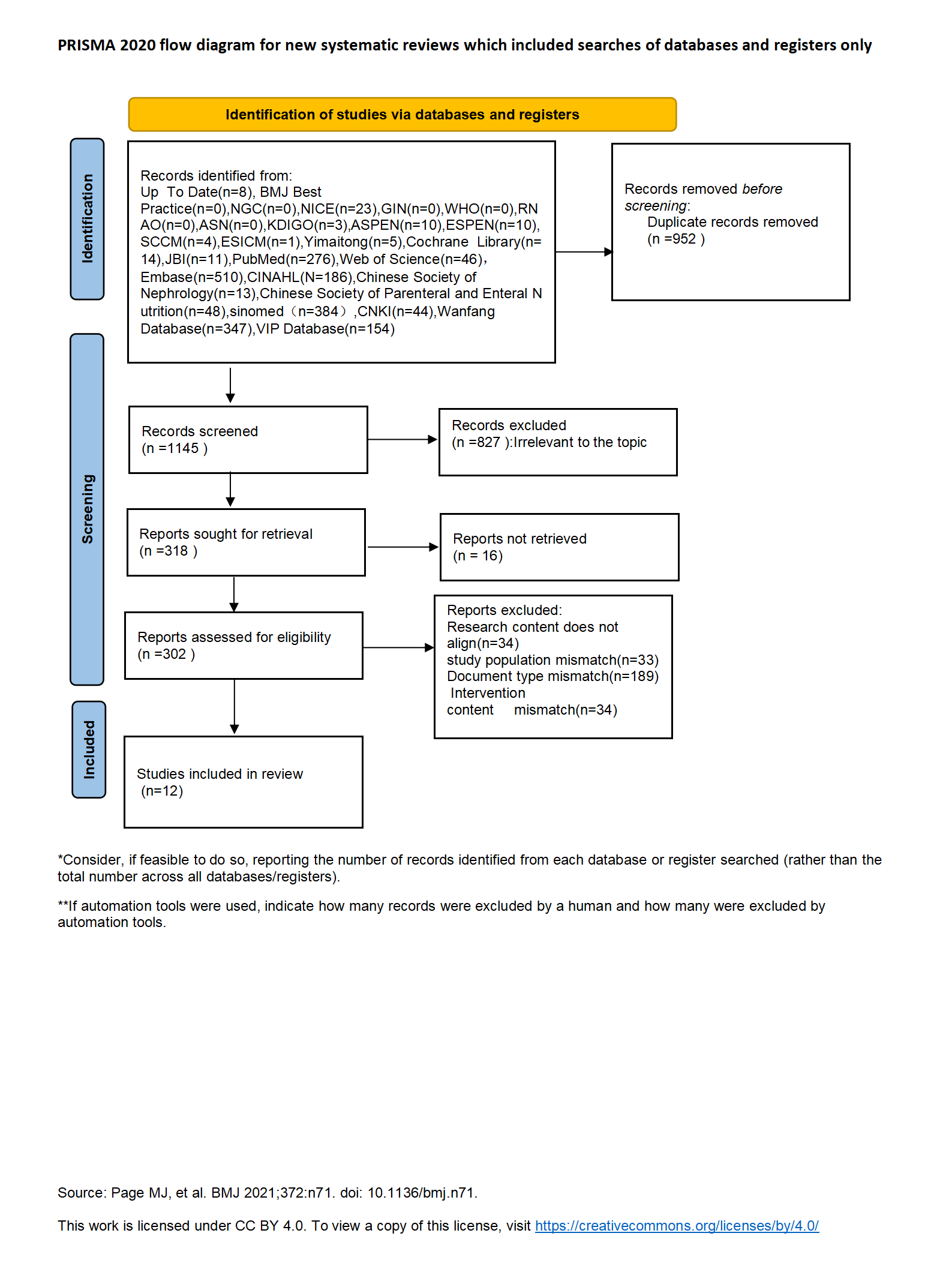

Supplement: Supplementary file 2 [file Image_1.tif]
